# Broad Scale Spatial Modelling of Wet Bulb Globe Temperature to Investigate Impact of Shade and Airflow on Heat Injury Risk and Labour Capacity in Warm to Hot Climates

**DOI:** 10.3390/ijerph20156531

**Published:** 2023-08-05

**Authors:** Andrew Hall, Ana Horta

**Affiliations:** Gulbali Institute, Charles Sturt University, Albury, NSW 2640, Australia; ahorta@csu.edu.au

**Keywords:** WBGT, global warming, heat injury, labour capacity, Australia, heat categories, relative humidity, wind speed, shade

## Abstract

While shade and air flow are recognised factors that reduce outdoor heat exposure, the level of reduction in terms of labour capacity at varying air temperature and humidity levels is poorly understood. This study investigated cooling effects on the commonly used heat index, wet bulb globe temperature (WBGT), and subsequent impact on labour capacity, for a range of air flow and shade conditions in warm to hot climates. We modelled heat exposure using a physics-based method to map WBGT for a case study region which experiences a range of heat categories with varying levels of health risks for outdoor workers. Continent-scale modelling confirmed significant spatial variability in the effect of various shade and wind speed scenarios across a range of real-world mid-summer daytime conditions. At high WBGTs, increasing shade or air flow for outdoor workers lowered heat exposure and increases labour capacity, with shade giving the greatest benefit, but cooling varied considerably depending upon underlying air temperature and humidity. Shade had the greater cooling effect; reducing incident radiation by 90% decreased WBGT by 2–6 °C depending on location. Wind had a lower cooling effect in the hottest regions, with a decreasing exponential relationship between wind speed and WBGT observed.

## 1. Introduction

Rising global temperatures are exposing more outdoor workers to heat levels that reduce their ability to safely undertake physical work [1,2,3]. In addition to air temperature, a key factor for determining bodily heat exchange is the evaporative potential of the air. A human body’s main mechanism for heat dissipation, sweating, is more effective at low humidities, as water evaporates more readily into drier air, increasing the rate of cooling through uptake of latent heat. A common meteorological metric, wet bulb temperature indicates potential evaporation in addition to air temperature [4,5]. It is therefore common for research addressing heat tolerance in humans undertaking physical activity to investigate the wet bulb temperature [6,7]. Wet bulb temperature (technically psychrometric wet bulb temperature, PWBT, which is measured inside a Stevenson screen, preventing solar and wind exposure, using a mercury thermometer that has its bulb wrapped in cloth kept moist by an associated water vessel), however, has limited efficacy in outdoor environments where the addition of wind and solar radiation are significant factors in heat exchange between a human body and the atmosphere. Air flow increases evaporation and enables more heat to be conducted away. Exposure to solar radiation directly transfers heat to a body. The wet bulb globe temperature (WBGT) metric [8] effectively integrates wind speed and radiation with air temperature and humidity. The International Standards Organisation adopted WBGT as the metric to describe heat exposure in 1982 [9,10], and it is applied in many outdoor work situations to assess heat exposure risk for persons conducting physical activity outdoors. Both the level of physical activity and WBGT are used to determine limits on physical work time and maintain a safe internal body temperature [11].

Heat mapping can incorporate modelled projections of future climatic conditions, based on Representative Concentration Pathways (RCP) developed by the Inter-governmental Panel on Climate Change. Different RCPs describe time series of atmospheric concentrations of greenhouse gases for particular socio-economic narratives. Scenarios for future climate conditions are referred to in terms of the additional downward forcing of radiated energy to the Earth’s surface (in Wm^−2^) in the year 2100, relative to 1986–2005. Intermediate RCP4.5 and high level RCP8.5 scenarios are regularly used in climate modelling studies. Psychrometric wet bulb temperature is commonly mapped for warming scenarios [6,7], with fewer studies mapping WBGT, as the implementation of reliable physics-based WBGT modelling is relatively complex in comparison to calculating PWBT. Willett and Sherwood [12] used a simplified WBGT [13], which considers neither wind nor solar variability to map WBGT across 15 dispersed regions under mid-range climate projections for the 2020s and 2050s. Kjellstrom et al. [14] applied a physics-based method of determining WBGT [15] to map WBGT at a low constant wind speed (1 ms^−1^) and NASA-sourced observed solar radiation for past (1975) and future (2050) scenarios for the hottest part of the year in SE Asia. Bitencourt et al. [16] used empirical equations [17] and observed wind speed to map estimated WBGT for Brazil under RCP4.5 and RCP8.5 for 2020 to 2100. Hall, Horta, Khan and Crabbe [18] built on these studies to map outdoor WBGT with the accurate Liljegren, Carhart, Lawday, Tschopp and Sharp [15] method, applying RCP4.5 and RCP8.5 scenarios for Australia. The Hall et al. [18] study restricted its base scenarios to two wind speeds and clear atmospheric conditions, limiting inferences that could be made about the effect of air flow and shade on WBGT. While the study generally demonstrated that a moderate wind speed significantly lowered WBGT compared to a light wind speed scenario, its effect at different base temperatures varied, with increasing wind speed delivering much less of a reduction in WBGT at higher baseline temperatures and humidities. By assuming clear atmospheric conditions, close to maximum possible solar loading was included in all modelled scenarios. The effect of shade on WBGT was therefore left uninvestigated. The present study investigates the effect on WBGT of varying wind speeds, including an observed wind speed scenario, and shade, by considering varying solar radiation scenarios.

In addition to expanding investigations into wind and shade effects on WBGT, this study takes a step into inferring reductions in labour capacity. By doing so, the economic effect on physical labour productivity of working under shade rather than being exposed to sunlight and of working with varying levels of air flow compared to calm conditions can be assessed for varying baseline WBGTs. There are recognised threshold values for physical work intensity levels corresponding to WBGT [19]. Time limits on continuous work at various workloads and WBGT categories are described by Patel et al. [20]. Full labour capacity is defined as the ability to perform continuous physical work for an 8-h period without being affected by heat stress. At light physical workloads (<233 W or activities such as walking or lifting less than 5 kg), recommended work times are only affected when heat category 5 (WBGT > 32.2 °C) is reached, reducing to 180 out of a possible 240 min. For moderate workloads (425 W), recommended work times at heat category 2 (WBGT 27.8–29.4 °C) and 5 (WBGT > 32.2 °C) are 150 and 70 out of 240 min, respectively. For heavy workloads (600 W, or activities such as lifting, carrying, digging), recommended work time is 70 min at heat category 1 (WBGT 25.6–27.7 °C) and 45 min at category 5.

A percentage reduction in full labour capacity is a metric developed by Dunne, Stouffer and John [21] based on an empirical relationship inferred from the amount of occupational work that can safely be performed by a healthy acclimatised individual without the risk of hyperthermic illness (Equation (1)).

(1) $$Labour\;Capacity(\%)=100-25\times \max{\left(0,WBGT-25\right)}^{2/3}$$

This metric was selected for the current study because of its ease of use when WBGT is known and its elegant simplicity relating it to established physical activity guidelines. The metric assumes an optimised work environment, which includes appropriate clothing that is consistent with the physical activity guidelines on which the parameters of the equation were derived. As a generalised metric, it is fitting to the study’s key aim of determining broad-scale economic impact of heat on labour capacity. Alternative metrics with greater specificity [22,23] to the effects of clothing, labour intensity and acclimation will likely perform better in guiding physical activity time limits in particular circumstances. The relationship defining the equation was informed by industrial and military physical activity guidelines employed by US organisations. It assumes that continuous 8 h of heavy labour (407–582 W in total energy production including metabolic heat and mechanical work, converted from the original publications units, kcal/h) can be safely undertaken up to a WBGT of 25 °C, but it is reduced to 50% for WBGT around 27.4 °C and to 25% when WBGT reaches 30 °C. A WBGT of 30 °C is also the threshold for performing continuous light labour. At a WBGT value of 32.2 °C, only 25% continuous light labour is physically possible, and no physical work is recommended for WBGT above 33 °C. It is possible to adjust labour capacity limits to other scenarios, e.g., for more specific work rates [23] or level of acclimation [22], but we used the general equation developed by Dunne et al. [21] for the broad range of climates being considered in this study, and a range of activity levels for acclimated workers.

The specific aims of the presented study were to quantify the effect of shade and wind speed on outdoor WBGT across a range of climates subject to heat categories that can lead to recommended restrictions in physical activity. The secondary aim was to make inferences about the level of reduction in lost labour capacity that can be realised through environmental modification by the provision of shade and air flow.

## 2. Materials and Methods

The spatial modelling framework developed by Hall, Horta, Khan and Crabbe [18], built around the Liljegren, Carhart, Lawday, Tschopp and Sharp [15] physics-based method of calculating WBGT, was employed as the main tool in this study. As the WBGT mapping exactly followed the method described in detail in this open-access refereed article [18] (although here with different datasets and scenarios) and because this method is complex with multiple steps and would repeat what has already been published previously, it is not presented here. Complex interrelationships between the relevant meteorological variables are features of any climate. For example, in hot climates with unlimited water, atmospheric latent heat uptake by evaporation moderates air temperature, whereas in hot dry climates sensible heat dominates and air temperatures are higher than in equivalent humid climates, resulting in a general inverse relationship between temperature and humidity. Complex relationships that vary spatially and seasonally exist for all four variables relevant to this study, i.e., temperature, humidity, windspeed and solar radiation. Rather than attempt to characterise these relationships, we used spatially interpolated Australia-wide climate data records, which implicitly include interactions of the relevant meteorological variables. While mostly arid, large regions of Australia have climates that can be characterised as humid tropical and humid temperate, with global warming expected to transition much of the continent to higher heat categories in the coming decades [18]. In addition to its climate being suitable for studies investigating the effect of heat stress on physical work capacity, there are reliable and readily available spatially and temporally extensive data for Australia’s climate.

Spatial data describing air temperatures and vapour pressure were acquired from the Australian Water Availability Project (AWAP) [24] repository [25,26], comprising daily interpolated digital climate maps with a cell size of 0.05 × 0.05 decimal degrees (approx. 5 km) for January maximum temperature and 3 pm vapour pressure (warmest part of the day at the warmest part of the year), January minimum temperature and 9 am vapour pressure (coolest part of the day at the warmest part of the year), and July’s maximum temperature and 3 pm vapour pressure (warmest part of the day at the coolest part of the year). The reference baseline period of 1986–2005 coincides with the baseline period of the Coupled Model Intercomparison Project Phase 5 (CMIP5) and enables broad comparisons with the majority of climate studies that use the same climate period.

Maps of modelled WBGT were produced for six wind scenarios: five spatially constant wind speeds, and one spatially heterogeneous observed averaged wind speed. The constant wind speeds were selected based on the Beaufort scale wind force boundaries (Table 1) which represent convenient recognisable wind speeds that increase at an exponential rate and are within the range of wind speeds relevant to Australia that are typically from 0.6 to 4.2 m/s in January and stronger in July when mean velocities are 5.6 m/s in northern and coastal southern Australia [18]. The average wind speed at each location was calculated from the daily average wind speed (m/s) provided by the CSIRO Land and Water Near-Surface Windspeed dataset [27], which consists of wind maps at different temporal and spatial resolutions created using the methodology in McVicar et al. [28]. We used the maps with 5 km spatial resolution and averaged the daily wind speeds for January and July for the baseline period (1986–2005).

To investigate the effect of shade on WBGT, we created solar radiation maps with fractionally reduced clear-atmosphere solar irradiance at mid-month for each of the three time scenarios: 15 January at 6 am, 15 January at 3 pm and 15 July at 3 pm. Note that the local time at all map cells was held consistent for each time scenario to ensure comparability at all longitudes, i.e., at all locations in each map the time is either 3 h after solar noon for 3 pm scenarios or 6 h before solar noon for the 6 am scenario. We first derived clear-atmosphere radiation scenario maps for the different time scenarios using the insolation function in the R module *insol* [30]. These baseline solar radiation maps were multiplied by 0.1 to 0.9 and 0.1 intervals to derive scenarios with equivalent shading of 90% to 10% of incoming solar radiation. For example, we used a value of total incident radiation equal to 10% of its original value to simulate the effect of 90% shade protection.

The resulting WBGT maps for the different wind and shade conditions were reclassified to the WBGT heat categories (Table 2) to compare changes between scenarios, using descriptive statistics and visual analysis. We also calculated labour capacity associated with each WBGT map by applying Equation (1) to every cell of the WBGT maps derived for different wind and shade conditions. The results varied between 0% (no physical work of any form recommended) and 100% (no recommended restriction on light, moderate or heavy labour).

## 3. Results

### 3.1. Effect of Wind Speed on WBGT

**Figure 1:**
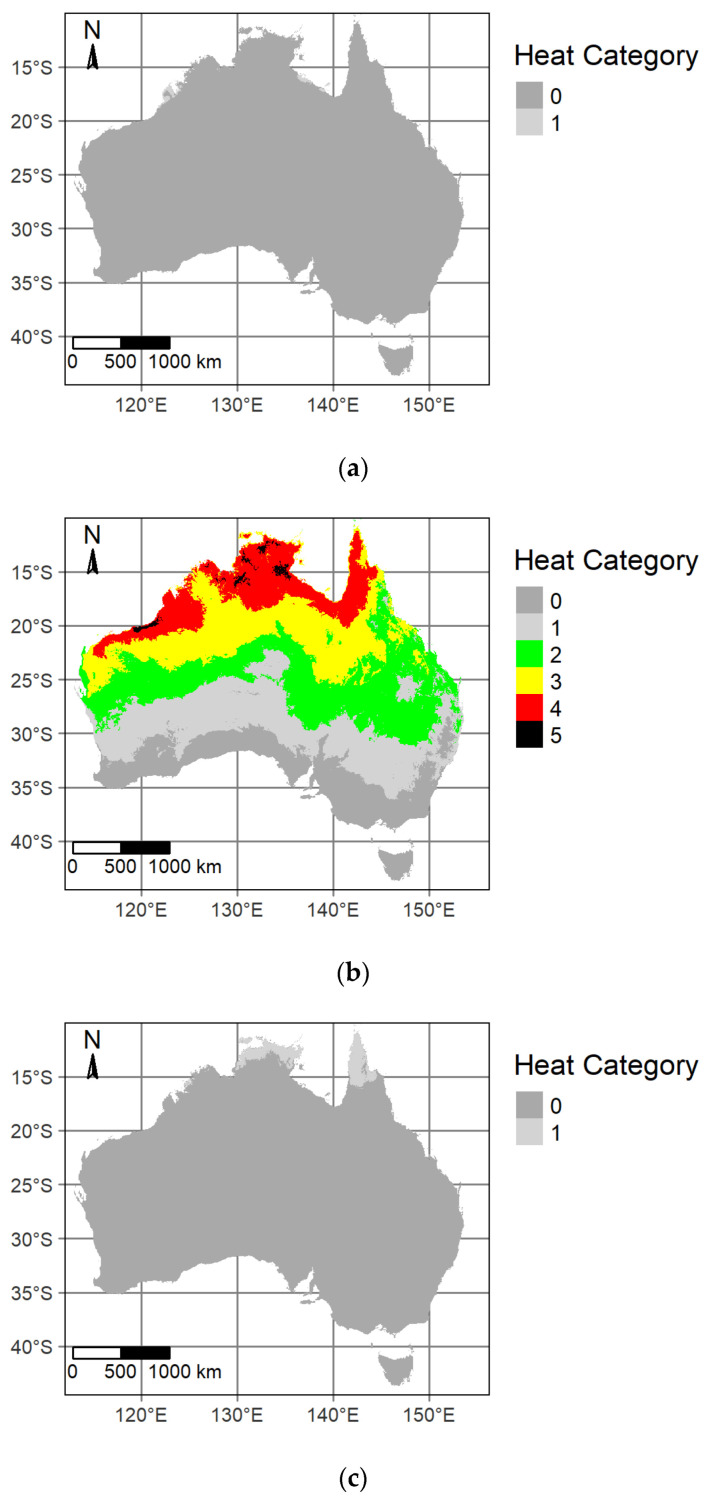
Mapped heat categories for (**a**) mid-January (summer) 6 a.m., (**b**) mid-January (summer) 3 p.m. and (**c**) mid-July (winter) 3 p.m. for the 1985–2005 climatology with averaged observed monthly wind speed and clear atmospheric conditions.

Spatial variability in heat categories, with the observed average monthly wind speed scenario, for mid-summer early morning, mid-summer daytime and mid-winter daytime is presented in Figure 1. Under baseline (1986–2005) daytime summer conditions, 58% of the country was classified into heat categories 2 to 5 (Table 2). The WBGT maps for both summer early morning and winter daytime scenarios indicated that only a relatively small area of the continent was classified to heat category 1 under winter daytime conditions with the remainder below the category 1 threshold of 25.6 °C. Given this result, the analysis of wind and shade on WBGT was performed for the mid-summer daytime scenario only, since this is the time of the year when the highest WBGT is likely to be experienced.

**Figure 2:**
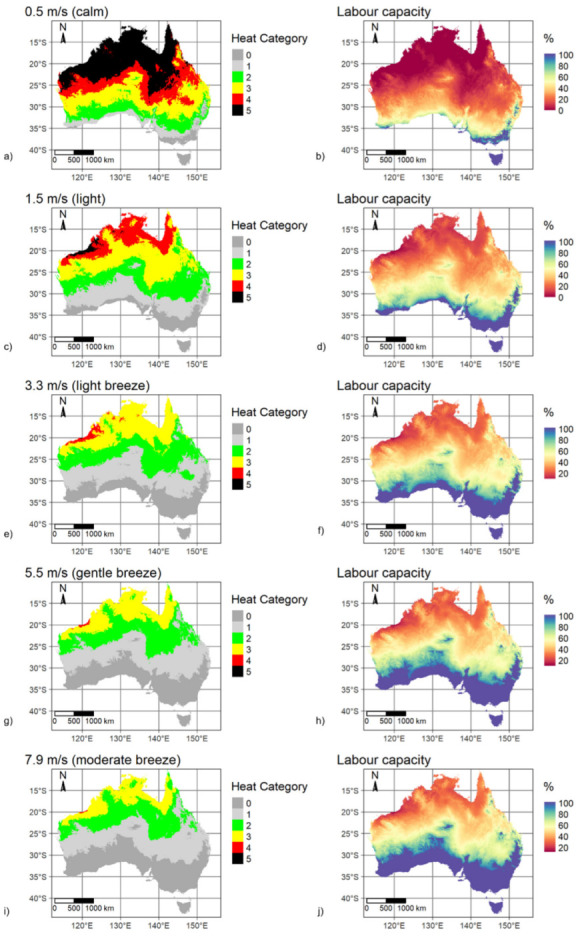
Mapped WBGT-derived heat categories and corresponding labour capacities (%), for baseline mid-summer daytime, for five spatially constant wind speeds. (**a**). heat category for 0.5 m/s wind speed; (**b**). labour capacity for 0.5 m/s wind speed; (**c**). heat category for 1.5 m/s wind speed; (**d**). labour capacity for 1.5 m/s wind speed; (**e**). heat category for 3.3 m/s wind speed; (**f**). labour capacity for 3.3 m/s wind speed; (**g**). heat category for 5.5 m/s wind speed; (**h**). labour capacity for 5.5 m/s wind speed; (**i**). heat category for 7.9 m/s wind speed; (**j**). labour capacity for 7.9 m/s wind speed.

Figure 2 presents maps of both heat categories and labour capacity for the summer daytime scenario with each of the five selected constant wind speeds applied. The highest WBGT (above 32.2 °C, heat category 5) was modelled with calm winds (0.5 m/s) for latitudes between 10 and 25° S, or most of the northern Australian region above the Tropic of Capricorn apart from eastern areas that are subject to prevailing easterly trade winds that bring cooler air from the Pacific Ocean. For the baseline climatology and calm wind conditions, Australia’s median WBGT was modelled as 31.2 °C (heat category 4), with minimum and maximum WBGT of 17.2 °C and 35.4 °C.

**Figure 3:**
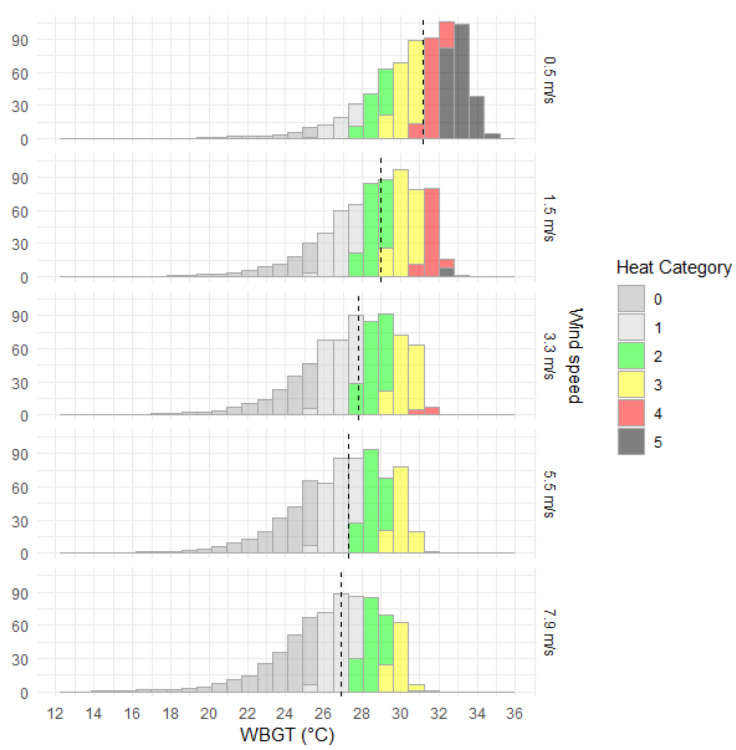
Distribution and median (dashed black line) of WBGT by area (×10,000 km^2^) of Australia for five different wind speeds for mid-January 3 p.m., 1986–2005 climatology and clear atmospheric conditions.

As expected, increasing wind speeds reduced modelled WBGT across all locations (Figure 2 and Figure 3). The most significant rates of change in WBGT with increasing wind speed were observed in the north, where most of the area north of 25° S was classified as heat categories 3 or 4 in light air (1.5 m/s) or light breeze (3.3 m/s) conditions. Increasing the wind speed from 0.5 m/s to 1.5 m/s decreased the median WBGT by 2.3 °C. Further increases in wind speed led to WBGT decreasing at a slower rate (1.0 °C from 1.5 m/s to 3.3 m/s, 0.6 °C from 3.3 m/s to 5.5 m/s and 0.3 °C from 5.5 m/s to 7.9 m/s). In more detailed analysis (results not presented), we examined the rate of change in WBGT for wind speed increases at 0.1 m/s increments from 0.5 to 1.5 m/s, revealing a clear decaying exponential relationship of a decreasing rate of change in WBGT for increasing wind speed.

The cooling effect of increasing wind speed is clear from Figure 2, with the total area classified as heat categories 0 or 1 approximately three times larger under light to moderate breeze conditions compared to calm conditions. Similar spatial arrangements (patterns) in WBGT variability were observed for all wind speed scenarios, indicating there was no underlying location-dependent processes to engender spatially variable rates of change in WBGT with respect to increasing wind speed. In the calm (0.5 m/s) summer daytime scenario, labour capacity was modelled to be less than 20% for most of the continent (Figure 2). Labour capacity significantly increased (to greater than 50%) for wind speeds above 1.5 m/s, but with northern areas maintaining low labour capacities despite higher wind speeds.

### 3.2. Effect of Shade Conditions on WBGT

**Figure 4:**
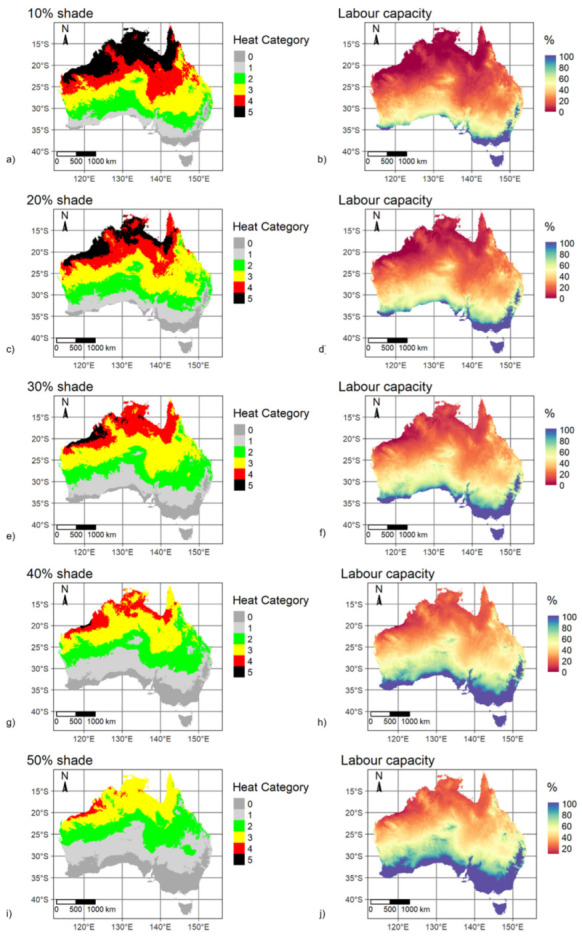

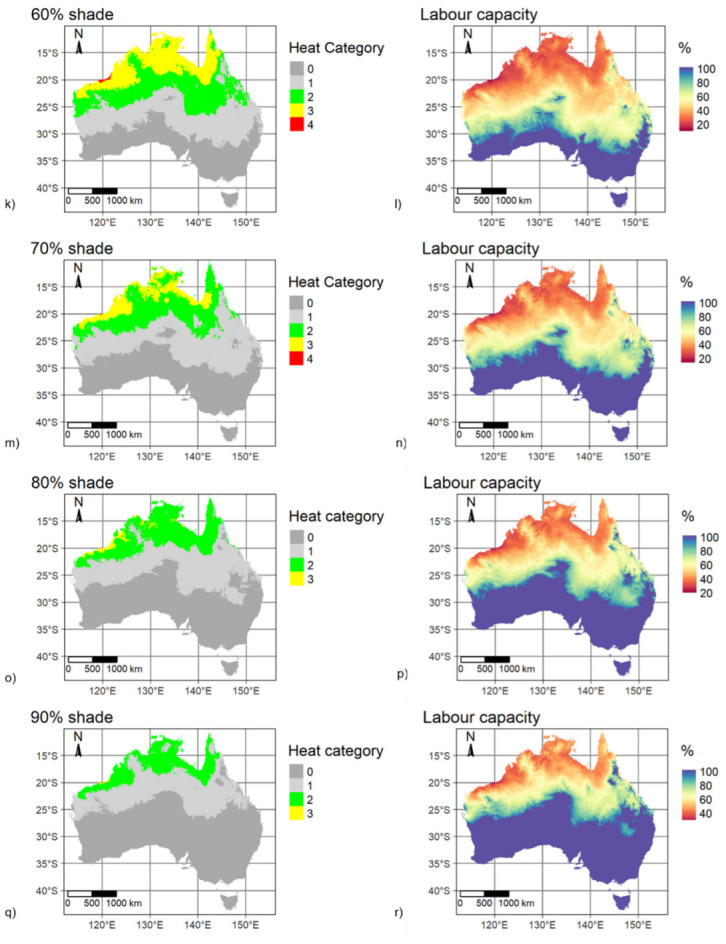
Mapped WBGT-derived heat categories and corresponding labour capacities (%) for baseline mid-summer daytime, calm wind (0.5 m/s) and clear atmosphere with decile shades applied. (**a**). heat category for 10% shade; (**b**). labour capacity for 10% shade; (**c**). heat category for 20% shade; (**d**). labour capacity for 20% shade; (**e**). heat category for 30% shade; (**f**). labour capacity for 30% shade; (**g**). heat category for 40% shade; (**h**). labour capacity for 40% shade; (**i**). heat category for 50% shade; (**j**). labour capacity for 50% shade; (**k**). heat category for 60% shade; (**l**). labour capacity for 60% shade; (**m**). heat category for 70% shade; (**n**). labour capacity for 70% shade; (**o**). heat category for 80% shade; (**p**). labour capacity for 80% shade; (**q**). heat category for 90% shade; (**r**). labour capacity for 90% shade.

**Figure 5:**
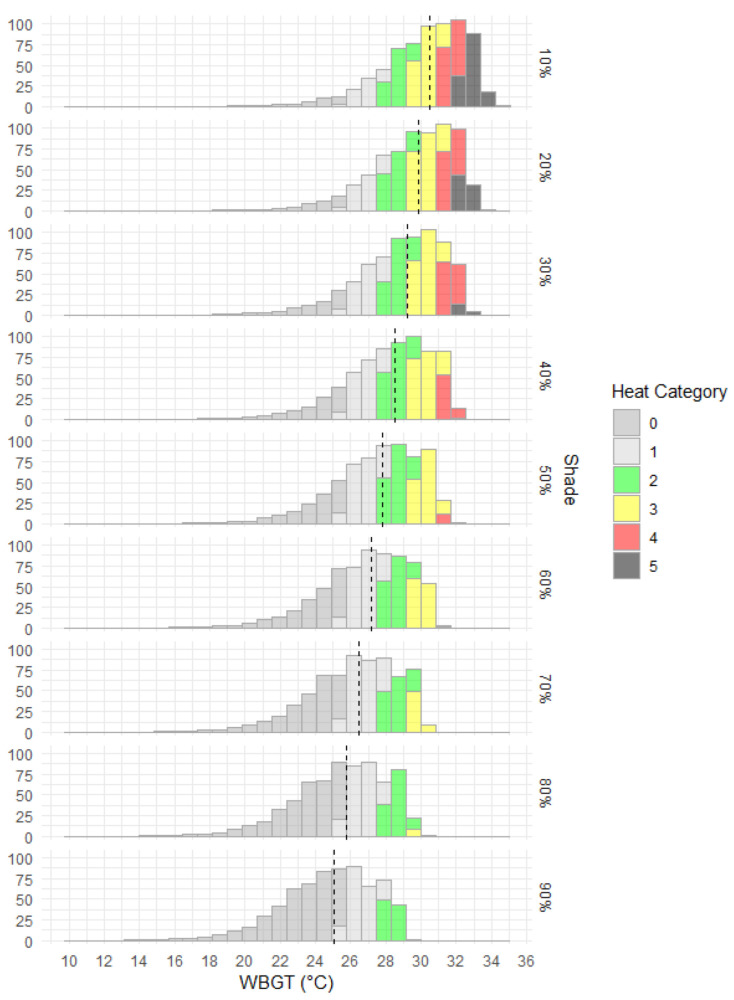
Distribution and median (dashed black line) of WBGT by area of Australia (×10,000 km^2^) for nine different shade levels for mid-January 3 p.m., 1986–2005 climatology and (otherwise) clear atmospheric conditions.

Nine scenarios that considered 10% to 90% reductions in solar radiation using baseline daytime summer calm-wind (0.5 m/s) conditions are mapped in Figure 4 with corresponding WBGT distributions in Figure 5. The spatial pattern in labour capacity is aligned with WBGT spatial variability, with most of the country having labour capacity above 70% when shade is greater than 40%. Continent-wide median WBGT decreased by 0.7 °C for each subsequent 10% increase in shade. Compared to the 10% shade scenario, median WBGT was reduced by 2.7 °C with 50% shade and 5.5 °C with 90% shade (Figure 5). Progressing through increasing levels of shade, at 40% shade there is a clear change point with a significant reduction in areas classified as heat categories 5 and 4 and an increase in areas classified as 0 and 1 (Figure 4). This shade analysis only considered direct solar radiation and assumed a clear atmosphere. Indirect solar radiation (i.e., reflected) and terrestrial radiation will potentially raise total incoming radiation. High-albedo environments (land surfaces with relatively high levels of reflectivity), in particular, can especially increase radiation loads. In addition, atmospheric transmissivity can vary considerably even in apparent cloud-free conditions to decrease total radiation loading at the Earth’s surface. Radiation regimes are further complicated by atmospheric and albedo spatial and temporal variability, especially due to changing moisture content. While specific cases for determining physical work time limits should consider radiation loading more accurately, for generalised baseline WBGT the simplified approach used in this study would unlikely have a major effect on calculated labour capacity.

**Figure 6:**
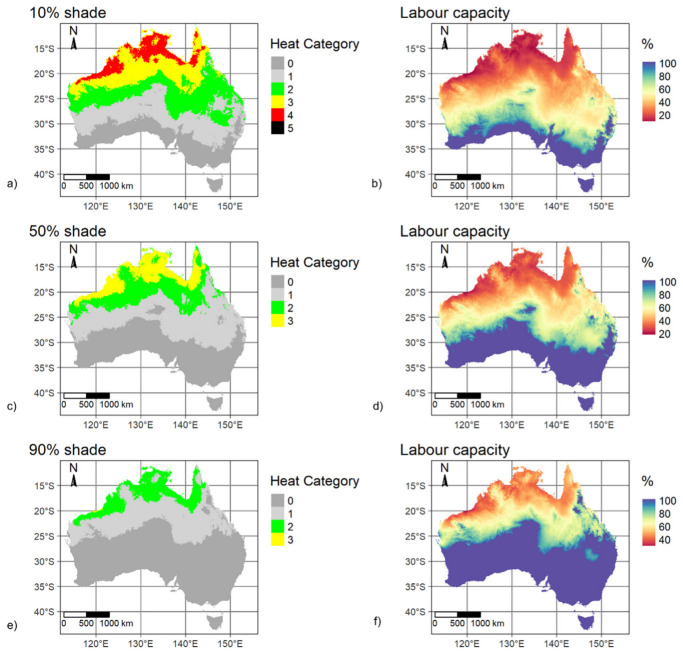
Mapped WBGT-derived heat categories and corresponding labour capacities (%) for baseline mid-summer daytime, monthly average observed wind and shade of 10%, 50% and 90% of equivalent clear atmosphere. (**a**). heat category for 10% shade; (**b**). labour capacity for 10% shade; (**c**). heat category for 50% shade; (**d**). labour capacity for 50% shade; (**e**). heat category for 90% shade; (**f**). labour capacity for 90% shade.

**Figure 7:**
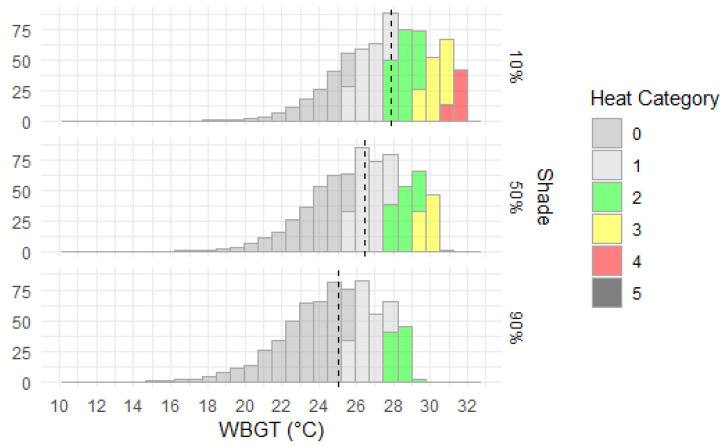
Distribution and median (dashed black line) of WBGT by area of Australia (×10,000 km^2^) for 10%, 50% and 90% shade levels for mid-January 3 p.m., 1986–2005 climatology and (otherwise) clear atmospheric conditions with monthly averaged observed wind speeds.

We also compared WBGT variability for baseline average wind conditions during daytime summer and three shade protection scenarios: 10%, 50% and 90% (Figure 6 and Figure 7). The results confirm that WBGT is reduced as shade increases, although, under average wind conditions, the WBGT median with 10% shade is equivalent to the WBGT median under calm wind (0.5 m/s) and 50% shade protection. In terms of WBGT spatial variability, the areas classified as heat categories 4 or 5 have a small extent compared to the other categories, are restricted to the most northern areas, and only occur for the 10% shade scenario. So, under typical wind conditions for daytime summer, labour capacity is only significantly restricted in these northern areas with low shade. Reducing incident radiation by 90% decreased WBGT by 2–6 °C depending on location, with the rate of cooling linearly related to increasing shade. The effect of wind speed on cooling was spatially variable, with a distinctly lower level of cooling in the hottest northern areas in response to increased wind speed. Further increases to wind speed led to less cooling, with an observed logarithmic relationship between wind speed and WBGT, whereby the rate of decrease in WBGT with respect to increasing windspeed was lower at high WBGTs.

## 4. Discussion

Previous studies investigating WBGT have assumed indoor conditions or constant wind speed and solar radiation settings [12,31], but by including potential radiation loads at various levels of shade, heat exposure levels for outdoor workers have been demonstrated in this study. The results clearly demonstrated a significant cooling effect of shade. Our modelling demonstrated that in mid-summer at 3 pm, a 50% reduction in solar radiation reduced WBGT by on average 2.7 °C and, for a 90% reduction in solar radiation, WBGT was on average 5.5 °C lower, which in turn led to significantly improved labour capacities. This compares similarly to findings by Honjo et al. [32] that stressed the importance of time of day and shadows in determining thermal comfort and by Lauwaet et al. [33] where WBGT was significantly reduced within forested environments. Provision of shade to otherwise exposed outdoor workers would have a significantly positive impact on physical labour productivity. In addition, through the production of a series of scenarios with varying levels of shade, which reduced modelled solar radiation by 10 to 90%, the level of reduction in WBGT by providing shade to outdoor workers was determined. In modelled scenarios where close to full shade was applied (90% shade), WBGT was consistently significantly lower compared to clear atmospheric conditions.

For the WBGT range modelled for Australian summer daytime corresponding with heat categories 1 to 5, this study’s investigation of the effect of varying wind speed on WBGT has confirmed a relationship between wind speed and lower WBGT, whereby the rate of change in cooling decreases at higher wind speeds. While a cooling effect of moderate wind speeds, compared to calm conditions, has been previously demonstrated by investigating two wind speed scenarios [18], and physically observed in specific case studies [15,34], this study’s systematic analysis of six wind speeds has confirmed a decreasing logarithmic relationship between WBGT and wind speed. A further significant output of the present study is WBGT scenarios that incorporate observed wind speed. A key scenario in the present study applied observed mean monthly wind speed to produce a more realistic map of heat categories for Australia. The observed wind speed scenario, which otherwise had the same parameters as a previous study [18] so as to be directly comparable, produced a map with greater WBGT than the 3 m/s scenario but not lower than the 0.5 m/s scenario.

Analysis of the WBGT maps produced in the present study shows that the level of reduction in WBGT by increasing wind speed at high extant WBGTs is less than that under conditions with lower extant WBGTs. At high WBGTs, wind speed has a reduced cooling effect; e.g., with air temperature at 40 °C and relative humidity of 50%, WBGT is minimally reduced by increasing wind speed [15]. This is because any heat flow through conductance will be from the air to a body when the surface temperature is less than the air temperature, counteracting any evaporative cooling, which would be relatively low at high levels of humidity. Air flow equivalent to a light breeze or more was found to decrease WBGT but by a lesser amount than that provided by shade, with further decreases in WBGT for stronger winds, but with diminishing rates of change for stronger winds. At high baseline temperatures and humidities (WBGT > 29 °C), increasing wind speed had little effect on reducing modelled WBGT.

The assessment of impact of WBGT levels for a baseline Australian climatology demonstrated that labour capacity is significantly reduced in outdoor locations across much of the Australian continent in summer, which is consistent with modelled worldwide trends for reduced work capacity in hot areas worldwide and consequent social and economic impacts [35]. Under the baseline summer conditions with a clear atmosphere and calm winds, approximately 15,00,000 km^2^ of Australia has heat conditions at which physical activity would be severely limited (0% labour capacity based on the Dunne, Stouffer and John [21] equation), for typical daytime conditions in mid-summer. This is consistent with findings of Takakura et al. [36] whereby, due to increasing WBGTs, a shift in working time will be required for outdoor work in Japan in the near future. The modelling used average January humidities and temperatures, and temporal variability in labour capacity due to daily variability in WBGT was not considered. A general inverse relationship between air temperature and relative humidity at any one location [37] would likely moderate the highest WBGT that might otherwise be suggested if both temperature and humidity are both set at above average. More modelling using detailed daily temperatures and humidities is therefore required for a full analysis of observed WBGT in Australia.

Modelled low summer daytime labour capacities across much of the continent for the baseline 1986–2005 period in conjunction with projected future WBGTs under climate change scenarios indicate that labour capacities across much of Australia are expected to decline for larger areas and greater proportions of the year, which is concomitant with physiological modelling for future Australian heat-exposure conditions determined by Hunt, Brearley, Hall and Pope [11]. This outcome is similar to that demonstrated for population centres of Brazil, which are subject to increasing heat categories comparable to Australia, increasing probability of heat stress across Brazil in coming decades with distinct spatial variation in rates of change [16,38]. Control of heat risk injury will therefore become more important for outdoor workers, such as personnel in emergency response, defence, agriculture, construction and mining. Many relatively cooler regions of Australia are on the threshold of WBGT temperatures that, with expected global warming in the coming decades, will more frequently be affected by WBGTs that exceed heat categories to restrict recommended physical activity.

## 5. Conclusions

By modelling a large case study area with extensive climate records at high spatial resolution, the effect of windspeed and shade on WBGT under varied scenarios was explored. While wind speed is widely acknowledged as having a cooling effect, its impact on reducing heat exposure at high humidities and temperatures is reduced as wind speed increases in a decreasing logarithmic relationship. For January in Australia north of 25° S, increasing the wind speed from 0.5 m/s to 1.5 m/s decreased the median WBGT by 2.3 °C, but increasing wind speed from 5.5 m/s to 7.9 m/s led to WBGT decreasing by only 0.3 °C. The effect on WBGT of various levels of shade was also explored across the case study region to determine shade and cloud level impacts on labour capacity. There was a clear linear reduction in WBGT for increasing levels of shade. Across the study area, with each subsequent 10% reduction in maximum solar exposure that would otherwise be experienced under clear atmospheric conditions, continent-wide median WBGT decreased by 0.7 °C.

Through an assessment of mapped heat categories and labour capacity for varying levels of reduced solar radiation and increased wind speeds, this study has demonstrated the impact of simple modification of outdoor working environments. Mapped WBGT is directly comparable to mapped labour capacity, with reductions in labour capacity starting at WBGT of 25 °C and reducing to 0% labour capacity at WBGT of 33 °C. Increasing both shade and air flow significantly lowers WBGT that in turn leads to substantial reductions in otherwise lost labour capacity. With continuing global warming increasing heat injury risk, knowledge of the impact of environmental modification to keep workplace temperatures within a safe range can help to maintain worker health and reduce deleterious economic impacts.

## Figures and Tables

**Table 1:** Constant wind speeds modelled in this study and equivalent Beaufort wind scale [29] force boundaries. The wind speeds modelled in this study correspond with the lower boundary of wind speed ranges of Beaufort wind scale force 1 to 5.

Wind Speeds Modelled in This Study (m/s)	Wind Force	Description	Specifications
0.5	1	Light air	Direction shown by smoke drift but not by wind vanes. Sea rippled
1.5	2	Light breeze	Wind felt on face; leaves rustle; wind vane moved by wind. Small wavelets on sea
3.3	3	Gentle breeze	Leaves and small twigs in constant motion; light flags extended. Large wavelets on sea
5.5	4	Moderate breeze	Raises dust and loose paper; small branches moved. Small waves, fairly frequent white horses
7.9	5	Fresh breeze	Small trees in leaf begin to sway; crested wavelets form on inland waters. Moderate waves, many white horses

**Table 2:** WBGT heat categories (United States Department of the Army, 1980) with conversion to Celsius scale from original Fahrenheit categorisation.

Heat Category	WBGT (°C)
1	25.6–27.8
2	27.8–29.4
3	29.4–31.1
4	31.1–32.2
5	>32.2

## Data Availability

The data presented in this study are available upon reasonable request from the corresponding author.
